# A stroma-corrected ZEB1 transcriptional signature is inversely associated with antitumor immune activity in breast cancer

**DOI:** 10.1038/s41598-019-54282-z

**Published:** 2019-11-28

**Authors:** C. James Block, Gregory Dyson, Ion John Campeanu, Donovan Watza, Manohar Ratnam, Guojun Wu

**Affiliations:** 1 Barbara Ann Karmanos Cancer Institute, Department of Oncology, Wayne State University School of Medicine, 4100 John R, Detroit, MI USA; 2 MD/PhD Program, Wayne State University School of Medicine, 320 E Canfield St., Detroit, MI USA

**Keywords:** Cancer genomics, Data mining, Gene regulatory networks

## Abstract

The epithelial-to-mesenchymal transition (EMT) is an essential developmental process which can be hijacked by cancer cells, leading to enhanced metastasis and chemoresistance in experimental models. Recent studies have linked gene expression of EMT-associated gene signatures to increased inflammatory immune response in multiple cancer types. However, these studies did not account for the potential confounding effects of gene expression by tumor-infiltrating mesenchymal stromal cells. In this study, we comprehensively dissect the associations between multiple EMT transcription factors and EMT markers with stromal and immune tumor infiltration. We find that EMT-related genes are highly correlated with intratumoral stromal cell abundance and identify a specific relationship between stroma-corrected *ZEB1* expression and decreased immune activity in multiple cancer types. We derive a stroma-corrected *ZEB1*-activated transcriptional signature and demonstrate that this signature includes several known inhibitors of inflammation, including *BMPR2*. Finally, multivariate survival analysis reveals that *ZEB1* and its expression signature are significantly associated with reduced overall survival in breast cancer patients. In conclusion, this study identifies a novel association between stroma-adjusted *ZEB1* expression and tumor immune activity and addresses the critical issue of confounding between EMT-associated genes and tumor stromal content.

## Introduction

The epithelial-to-mesenchymal transition (EMT) is a critical process in early development by which embryonic cells migrate to properly form new tissues^1^. Accumulated experimental evidence has suggested that EMT is pathologically re-activated in epithelial cancers, leading to an increased propensity for chemotherapeutic resistance, tumor recurrence, and distal metastatic progression^1,2^. Breast cancer has served as one of the primary models for studying the role of EMT in cancer progression. It has been demonstrated both *in vitro* and *in vivo* that breast cancer cells that undergo EMT gain stemlike features, increased chemoresistance, and an enhanced ability to invade local tissues and colonize distant organs^3^. However, the role of EMT in patient outcomes remains controversial.

An EMT can be provoked by several signaling pathways which converge on the activation of a group of EMT-promoting transcription factors (EMT-TFs), including members of the SNAI, TWIST, ZEB and FOX transcription factor families^4^. This diverse group of transcription factors can directly repress the expression of critical epithelial adhesion proteins, like E-cadherin (*CDH1*), and upregulate mesenchymal cytoskeletal and extracellular matrix components^1,4^. Mechanistically, ectopic expression of any individual EMT-TF is sufficient to induce an EMT. Despite this commonality, the relationship between different EMT-TFs is complex and not fully understood. Developmental studies have demonstrated EMT-TFs have distinct roles during embryogenesis^5^. In parallel, recent studies have demonstrated that EMT-TFs have different proclivities for promoting cancer chemoresistance and metastasis^6,7^. Despite these differences, few studies have examined multiple EMT-TFs simultaneously at either the functional or genomic level^5^.

Experimentally, EMT has been shown to suppress the immune response in multiple cancer types^8,9^. Interestingly, EMT can be induced by inflammatory signals^10^. These post-EMT cells can then act to suppress inflammation within tumors^10^. Several recent studies of gene expression data from patient-derived tumor samples have attempted to evaluate the relationship between EMT and antitumor immune activity, with conflicting results. Multiple groups have linked the expression of EMT-associated genes to increased immune cell infiltration and increased intratumoral inflammation^11,12^. Conversely, a study in lung squamous and adenocarcinoma found that an EMT gene signature was associated with decreased T-cell infiltration and increased expression of immunosuppressive cytokines^13^.

Other studies have attempted to use similar EMT-associated gene signatures to predict survival outcomes in patients^14^. However, these prior inquiries did not account for potential confounding by stromal cell infiltration into the tumor sample^15^. The patient-derived tumor sequencing data used in these studies were generated by bulk tumor sequencing, which generates an expression measurement for each gene averaged across all of the cells present within the sample. Consequently, the presence of both mesenchymal stromal cells and immune cells can contribute to the overall gene expression signal and confound evaluations of the contribution of a gene or gene signature to both tumor biology and clinical outcomes. As EMT-associated genes are highly expressed by mesenchymal cell types, the potential for confounding is substantial. For example, a recent study demonstrated that EMT and stromal cell signatures can confound one another and in general are associated with an altered response to immunotherapy in urothelial carcinoma^16^.

An example of this potential confounding effect is shown in Supplemental Fig. 1. Gene X is a gene that is associated with the mesenchymal phenotype, being expressed at a higher level in fibroblasts and EMT-like cancer cells than in epithelial cancer cells (Supplemental Fig. 1A). Two hypothetical tumor samples, sample A and sample B, have an equal proportion of fully epithelial cancer cells to cancer cells undergoing EMT (5:1). However, sample B contains a higher number of infiltrating mesenchymal stromal cells. As a result, the estimated gene expression of the mesenchymal-associated gene Gene X will be higher in sample B than sample A, even after normalization (Supplemental Fig. 1B). If the effect of stromal cell abundance is not considered, a researcher may make spurious conclusions about the associations between the expression of Gene X and both tumor biology and patient outcomes. However, gene signature-based deconvolution methods can be used to estimate the abundance of stromal cells present within the tumor and then correct the gene expression levels to represent the cancer-specific expression of Gene X^17^. Thus, it is critical to account for tumor purity in any study that attempts to associate tumor gene expression to clinical or pathologic characteristics.

In this study, we used multiple gene signature-based methods to evaluate the association between the expression of EMT-TFs and EMT marker genes with both stromal and immune cell content in tumor samples. These analyses revealed critical differences between different EMT-TFs and markers, highlighting the complexity of EMT-associated processes. Our results demonstrated that the stroma-adjusted expression of one EMT-TF, *ZEB1*, has a unique inverse association with immune cell abundance and activity in multiple cancer types. As well-characterized EMT markers did not recapitulate this relationship, we derived a stroma-corrected, *ZEB1*-regulated gene signature with an integrative transcriptomic approach. The *ZEB1* gene signature showed similar associations with both stromal and immune cell abundance as *ZEB1* and contained several known repressors of the immune response, including the receptor *BMPR2*. Finally, a multivariate survival analysis demonstrated that *ZEB1* and members of the *ZEB1* gene signature are significant independent predictors of overall survival in breast cancer. Our results confirm the importance of considering the complicated cellular composition of tumors when performing gene association analyses. Importantly, this study demonstrates that stroma-corrected *ZEB1* transcriptional activity is associated with decreased abundance of tumor-infiltrating immune cells and decreased intratumoral inflammation, providing new insight into the role of *ZEB1* as a suppressor of antitumor immune activity.

## Results

### EMT-TFs exhibit distinct correlative relationships with stromal and immune cell abundance in primary tumor samples

**Figure 1:**
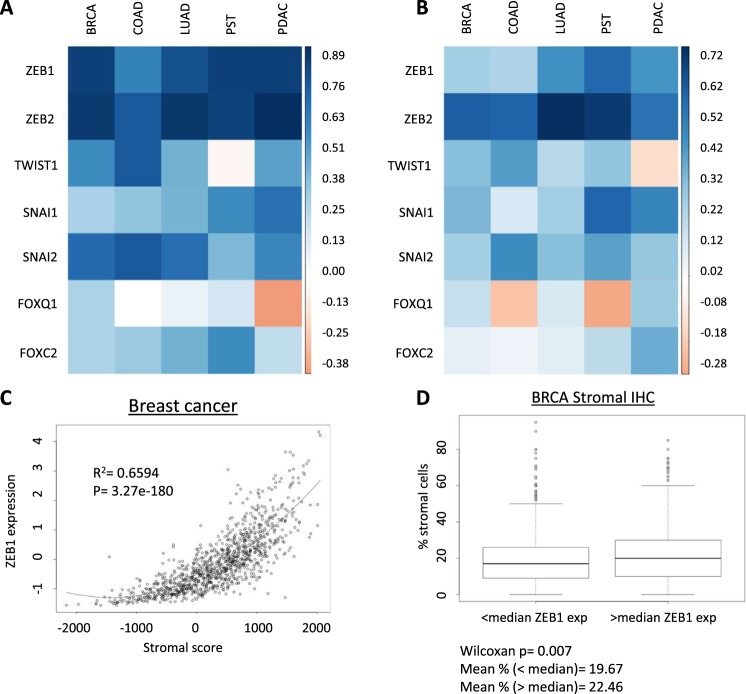
EMT-TFs are significantly positively correlated with stromal and immune cell abundance in multiple cancer types. (**A**) Heatmap illustrating Spearman correlation coefficients for selected EMT-TFs and ESTIMATE stromal score across five cancer types (breast (BRCA), colorectal (COAD), lung (LUAD), prostate (PST) and pancreatic (PDAC) adenocarcinoma. (**B**) Heatmap illustrating Spearman correlation coefficients for selected EMT-TFs and ESTIMATE immune score across same five cancer types. (**C**) Scatterplot illustrating correlation between ZEB1 expression and stromal score in BRCA dataset. Table shows correlation coefficients for both ER+ and ER− subsets. (**D**) Comparison of immunohistochemistry-estimated percentage of stromal cells between patients with less or greater than median ZEB1 (z score = −0.3026) mRNA expression (n = 1078 for all BRCA analyses).

As tumor-infiltrating stromal cells express high levels of mesenchymal-associated genes, we examined whether the expression of a set of well-characterized EMT-driving transcription factors was correlated with stromal cell infiltration in multiple cancer types. Normalized mRNA expression data from TCGA studies of breast, prostate, lung, colorectal and pancreatic adenocarcinomas was downloaded for *ZEB1*, *ZEB2*, *SNAI1*, *SNAI2*, *TWIST1*, *FOXQ1* and *FOXC2*^18,19^. These data were combined with stromal and immune cell abundance scores generated by the ESTIMATE approach, then evaluated for the Spearman correlation coefficient between both scores and the expression of each EMT-TF^20^. *ZEB1*, *ZEB2*, *SNAI1*, *SNAI2* and *FOXC2* exhibited consistent positive correlations with the ESTIMATE stromal score in all five cancer types (Fig. 1A, Supplemental Table 1). Of these, *ZEB1* (r = 0.60 to 0.84) and *ZEB2* (0.75 to 0.89) exhibited the strongest overall correlations. However, *ZEB2* is one of the 141 genes used to generate the ESTIMATE stromal score. Interestingly, *TWIST1* was not significantly correlated with stromal score in prostate cancer samples, while *FOXQ1* was not significantly correlated with stromal cell abundance in colorectal and lung adenocarcinoma. Also, *FOXQ1* was significantly inversely correlated with stromal score in pancreatic ductal adenocarcinoma. The distinct patterns of association of each EMT-TF demonstrate the unique expression profile of different EMT-TFs, highlighting the potential functional differences between these factors.

Next, this approach was repeated to determine the correlation between EMT-TFs and immune cell abundance. *ZEB1*, *ZEB2*, *SNAI1*, and *SNAI2* were significantly and positively correlated with the ESTIMATE immune score in all five cancer types examined (Fig. 1B). However, *FOXQ1*, *TWIST1* and *FOXC2* were either not significantly correlated or were modestly inversely correlated with immune score (Fig. 1B).

After removing ZEB2 from consideration, *ZEB1* was most significantly correlated with stromal infiltration in breast cancer (r^2^ = 0.65, p = 3.99 × 10^−180^) (Fig. 1C). To confirm this observation, the relationship between *ZEB1* mRNA expression and an immunohistochemistry-based estimation of the percent of stromal cell abundance was evaluated. As immunohistochemistry is a semi-quantitative approach for estimating cell content, samples were split into two groups by median *ZEB1* expression. *ZEB1* was significantly associated with stromal cell abundance (Wilcoxon p = 0.007), despite previous observations that IHC-based estimates of tumor stromal content are less sensitive than genomics-based approaches (Fig. 1D)^21^.

From these analyses, we concluded that several EMT-TFs are correlated with stromal cell abundance within tumors and that *ZEB1* has a particularly strong and reproducible association with tumor stromal content in breast cancer. Similarly, multiple EMT-TFs are positively associated with immune cell content before considering the potential effect of stromal cell abundance. This relationship may explain the prior reports of a positive correlation between EMT and immune activity.

### Stroma-corrected ZEB1 expression is inversely correlated with total immune cell abundance in primary tumor samples

**Figure 2:**
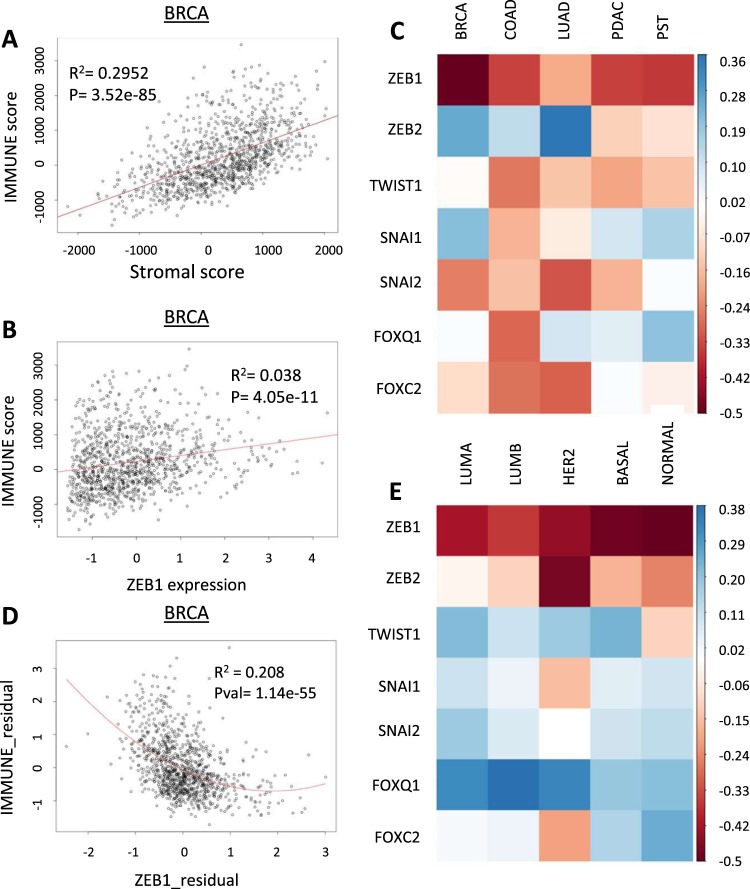
Stroma-corrected ZEB1 expression is inversely correlated with total immune cell abundance in tumor samples. (**A**) Correlation between ESTIMATE stromal and immune scores in BRCA dataset. (**B**) Correlation between ZEB1 expression and immune scores in BRCA. (**C**) Heatmap illustrating partial Spearman correlation coefficients of EMT-TFs with ESTIMATE immune score in five cancer types. (**D**) Residual plot of ZEB1 expression and ESTIMATE immune score, adjusted by stromal score. (**E**) Partial correlation coefficients for EMT-TFs and immune score by PAM50 subtypes of breast cancer.

To reproduce prior reports, we evaluated the association between immune and stromal cell infiltration. We found that stromal cell and immune cell abundance were highly correlated (Fig. 2A)^21^. We noted a discrepancy between the correlation values between EMT-TFs and stromal cell abundance when compared against immune cell abundance: In general, the correlation coefficients were much lower between EMT-TFs and immune cell content. For example, the coefficient of determination for the relationship between *ZEB1* and immune score in breast cancer was 0.038, much lower than the correlation between stromal and immune scores (Fig. 2B).

To correct for possible stromal cell confounding, the partial correlation coefficient was calculated for each EMT-TF with immune score. The partial correlation approach considers the correlations between two variables which are each highly correlated with a third variable, then corrects for the possible confounding effect of that third variable^22^. This analysis was repeated for each EMT-TF in each tumor type and plotted as a heatmap (Fig. 2C, Supplemental Table 2). As expected, correcting for stromal cell abundance significantly altered the relationships between EMT-TF expression and immune cell content. Uniquely, stroma-corrected *ZEB1* expression was significantly inversely correlated with total immune cell abundance in all five tumor types (Fig. 2C). *TWIST1*, *SNAI2* and *FOXC2* also showed moderate inverse correlations with immune cell content in four of five cancer types (Fig. 2C). The inverse relationship between *ZEB1* and immune cell content was strongest in breast cancer (partial r = −0.5, r^2^ = 0.208) (Fig. 2C,D). As breast cancer is a heterogenous disease comprised of multiple molecular subtypes, we re-evaluated this association by PAM50 subtypes. The PAM50 approach uses gene expression data to cluster breast cancer samples into five subtypes: Luminal A and B, Her2, Basal, and Normal^23^. PAM50 scores for the TCGA breast cancer cohort were obtained for a recent study^24^. Despite the differences between PAM50 subtypes, stroma-corrected *ZEB1* expression was consistently inversely correlated with immune cell abundance in all subtypes (Fig. 2E, Supplemental Table 3). However, *ZEB1* exhibited the strongest inverse associations in the basal and normal PAM50 samples (partial r = −0.492 and −0.504, respectively).

### Stroma-corrected ZEB1 expression is inversely associated with the abundance of multiple immune cell types and with immune cell recruitment to breast tumors

Our results demonstrated that the positive associations identified between EMT-TF expression and immune cell content were due to confounding by stromal cell abundance. By adjusting for stromal cell abundance, partial correlation analysis indicates a general trend towards an inverse association between EMT-TF expression and immune cell abundance in tumors.

**Figure 3:**
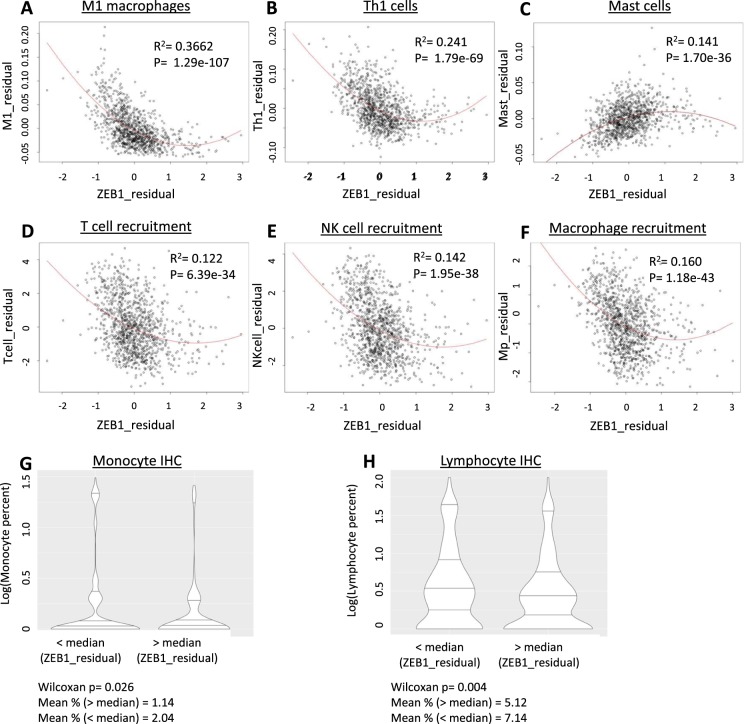
Stroma-corrected ZEB1 expression is inversely correlated with the abundance of multiple immune cell types and with immune cell recruitment to breast tumors. (**A**–**C**) Residual plot of ZEB1 expression and xCELL macrophage (**A**), Th1 cell (**B**) and mast cell (**C**) abundance corrected for stromal cell content. (**D**–**F**) Residual plot of ZEB1 expression and TIP-based estimation of T cell (**D**), natural killer cell (**E**) and macrophage (**F**) recruitment. (**G**) Immunohistochemistry measurement of monocyte percentage in tumor samples compared by samples with greater or less than median stroma-adjusted ZEB1 expression (median percentage 0%). (**H**) Immunohistochemistry measurement of lympocyte percentage in tumor samples compared by samples with greater or less than median stroma-adjusted ZEB1 expression (median percentage 1%).

Interestingly, stroma-corrected *ZEB1* expression showed a strong and reproducible inverse relationship with immune cell abundance, particularly in breast cancer samples. To investigate this association in more detail, the xCELL algorithm was used to generate estimations of the abundance of different cell types within breast tumor samples^25^. The partial correlation coefficient for each cell type with *ZEB1*, corrected for stromal cell abundance, was then generated (Supplemental Table 4). We observed that *ZEB1* was inversely correlated with multiple immune cell types, including M1 macrophages and Th1 cells (Fig. 3A,B). Both M1 macrophages and Th1 cells are known to promote inflammation and the antitumor immune response^26^. Th1 cells are known activators of antitumor macrophage activity, lending further credence to our results^26,27^. *ZEB1* was moderately positively associated with the infiltration of T-regulatory cells, well-characterized suppressors of the antitumor immune response (partial r = 0.177)^28^. Unexpectedly, *ZEB1* was also positively associated with mast cell abundance (partial r = 0.40, r^2^ = 0.141) (Fig. 3C). Mast cells are typically considered immunosuppressive within the tumor microenvironment, and the presence of mast cells has been associated with worse prognosis^29^. When we generated a correlation matrix for all 64 cell types in the breast cancer dataset, we found that mast cells were only modestly positively correlated with other immune cell types and were negatively correlated with Th1 cell abundance (Supplemental Fig. 2).

As several of the immune cell types which were inversely correlated with *ZEB1* are known to activate the immune response, we dissected the immune activity profile of the breast cancer tumor samples using the Tumor ImmunoPhenotype server algorithm (TIP)^30^. Like xCELL, TIP uses gene sets to evaluate immune activity in bulk tumor samples. However, instead of evaluating by cell type, TIP estimates the relative activity of different stages of the immune response, from tumor-antigen presentation to tumor cell killing by immune cells^30^. TIP scores were downloaded and compared with *ZEB1* expression by partial correlation analysis. Stroma-corrected *ZEB1* expression was significantly inversely correlated with five out of seven “steps” of the immune cycle: Antigen release, immune cell priming and activation, immune cell recruitment to the tumor, tumor infiltration, and cell killing (Table 1). *ZEB1* exhibited a modest positive correlation with cancer cell antigen presentation and was not correlated with cancer cell recognition by immune cells (Table 1). Of the immune cycle steps, stroma-corrected *ZEB1* expression was most significantly associated with immune cell activation and recruitment (Steps 3 and 4) (Table 1). Step 4 was then subset by recruitment of different immune cell types. By cell type, stroma-adjusted *ZEB1* expression was most significantly inversely associated with macrophage, T cell, and NK cell recruitment (Fig. 3D–F).

**Table 1:** Stroma-corrected ZEB1 expression is inversely associated with immune cell infiltration and activity in breast tumors.

Step of immune response	Description	Partial corr.	p.value
Step 1	Cancer Cell Ag Release	−0.1373	5.26E-06
Step 2	Cancer Ag Presentation	0.1469	1.08E-06
Step 3	Priming and Activation	−0.2222	1.10E-13
Step 4: Recruitment	T cell.recruiting	−0.3592	1.36E-34
	CD4 T cell.recruiting	−0.2060	6.22E-12
	CD8 T cell.recruiting	−0.3026	1.45E-24
	Th1 cell.recruiting	−0.2746	2.40E-20
	Dendritic cell.recruiting	−0.2695	1.27E-19
	Th22 cell.recruiting	−0.2518	2.95E-17
	Macrophage.recruiting	−0.4693	6.69E-61
	Monocyte.recruiting	−0.1986	3.57E-11
	Neutrophil.recruiting	−0.2568	6.67E-18
	NK cell.recruiting	−0.3840	1.11E-39
	Eosinophil.recruiting	−0.4318	8.16E-51
	Basophil.recruiting	−0.3382	1.26E-30
	Th17 cell.recruiting	−0.1572	1.78E-07
	B cell.recruiting	−0.1088	0.000317
	Th2 cell.recruiting	−0.3104	8.07E-26
	Treg cell.recruiting	−0.1810	1.71E-09
	MDSC.recruiting	−0.2294	1.67E-14
Step 5	Tumor infiltration	−0.1302	1.59E-05
Step 6	Recognition	−0.0379	0.210775
Step 7	Cell killing	−0.1947	8.56E-11

The results generated by TIP reproduced the results from the xCELL-based approach, increasing our confidence that stroma-corrected *ZEB1* expression is indeed associated with decreased immune cell abundance and antitumor immune activity in breast cancer. However, both methods are rely on GSEA-based approaches. As a confirmation that *ZEB1* expression was inversely associated with inflammatory activity in breast tumors, the partial correlations between stroma-adjusted *ZEB1* expression and the expression of nine pro-inflammatory cytokines (*CCL2*, *CCL4*, *IFNG*, *IL6*, *IL18*, *IL1A*, *IL1B*, *and MIF*, *TNF*) were calculated. When corrected for stromal abundance, *ZEB1* was significantly inversely correlated with all the cytokines examined (Supplemental Fig. 3A). *ZEB1* was most significantly correlated with decreasing expression of *IL-18* (partial r = −0.403), *MIF* (partial r = −0.394), *CCL2* (partial r = −0.367) and *CCL4* (partial r = −0.396). As these cytokines have been implicated in macrophage activation and polarization to the M1 phenotype, these data further support a role for *ZEB1* in the suppression of antitumor macrophage activation^31,32^.

As an alternative to transcriptomic data, we investigated the association between stroma-corrected *ZEB1* expression with immunohistochemistry-based measures of immune cell infiltration. When samples were split by median stroma-adjusted *ZEB1* expression, *ZEB1* was significantly associated with a lower percentage of monocytes (p = 0.026) and lymphocytes (p = 0.004) in primary tumor samples (Fig. 3G,H). Finally, we validated that the association between stroma-adjusted *ZEB1* expression and immune infiltration was not specific to the TCGA BRCA cohort by repeating these analyses in the METABRIC breast cancer expression dataset^33^. ESTIMATE stromal and immune scores were generated with the ESTIMATE R package and the prior analyses were repeated (Supplemental Data 1)^20^. Unadjusted *ZEB1* expression was significantly correlated with stromal score (r = 0.54) (Supplemental Fig. 3B) When *ZEB1* expression was adjusted by stromal score, *ZEB1* was significantly inversely correlated with immune score (partial r = −0.28) (Supplemental Fig. 3C).

### EMT marker genes exhibit distinct patterns of association with tumor stromal and immune cell abundance

Together, our results strongly indicated that stroma-corrected *ZEB1* expression is associated with a reduced antitumor immune response in breast cancer. Specifically, *ZEB1* is inversely correlated with both the abundance of pro-inflammatory and immune-activating cells and with measures of immune activation and immune cell recruitment. This finding suggested two possible explanatory hypotheses: (1) That *ZEB1* is a marker for EMT, and that EMT is driving the inverse association, or (2) that genes specifically under the regulation of *ZEB1* are responsible for this relationship.

**Figure 4:**
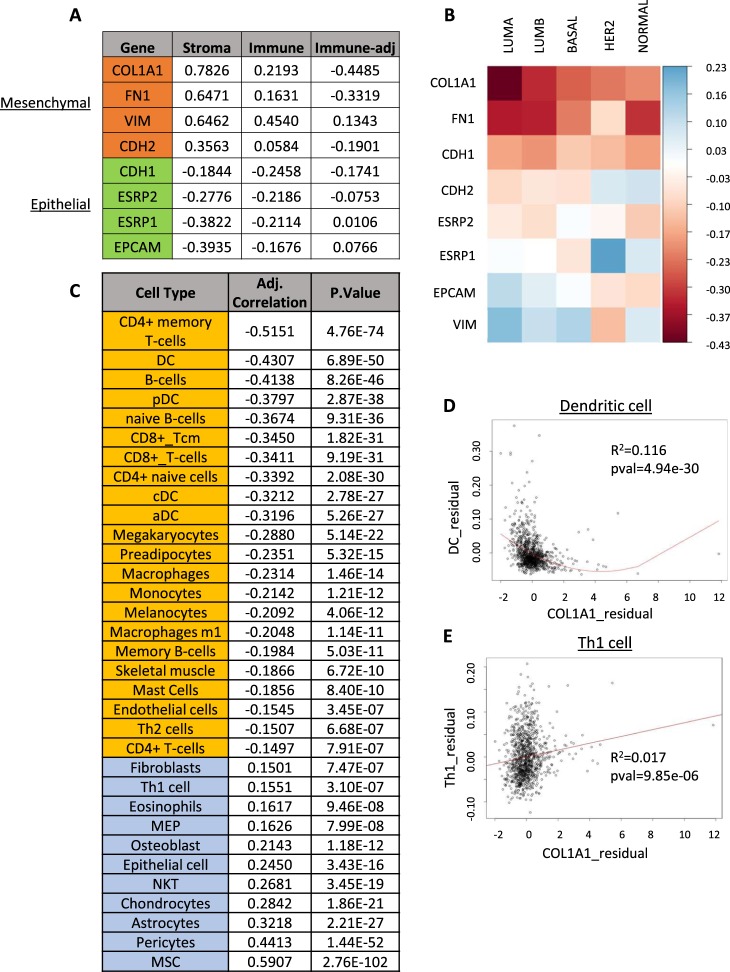
Association of EMT markers with stromal and immune cell abundance in breast tumor samples. (**A**) Correlation of selected EMT marker genes with stromal, immune, and stroma-adjusted immune abundance. Mesenchymal markers are highlighted in orange, and epithelial genes are highlighted in green. (**B**) Heatmap representing partial correlation coefficients for EMT marker genes and immune cell abundance, adjusted for stromal cell content. (**C**) Partial correlation coefficients for COL1A1 and xCELL cell types adjusted for stromal content. (**D**,**E**) Residual plot of adjusted COL1A1 expression and dendritic cell (**D**) and Th1 cell (**E**) content.

To investigate the possibility that the association we observed between stroma-corrected *ZEB1* expression and anti-tumor immune activity were due to its role as a marker for EMT, we examined the relationship of a representative sample of well-characterized EMT maker genes with both stromal and immune cell abundance. Based on a literature review, the genes collagen 1a1 *(COL1A1)*, fibronectin *(FN1)*, vimentin (*VIM*) and N-cadherin (*CDH2*) were selected as mesenchymal markers, while E-cadherin (*CDH1)*, epithelial splicing regulatory protein 1 and 2 (*ESRP1*,*2)*, and epithelial cell adhesion marker *(EPCAM)* were selected as epithelial markers^34^. As expected, the mesenchymal marker genes were all positively correlated with stromal cell abundance, while the epithelial markers were inversely correlated (Fig. 4A). The same pattern was observed for the association between gene expression and immune cell content (Fig. 4A). A more complicated picture emerged when the partial correlation between each gene and immune cell abundance, adjusted for stromal cell abundance, was calculated. *COL1A1* and *FN1* exhibited strong inverse correlations with immune cell abundance, as expected (Fig. 4A). However, *CDH2* exhibited a much lower inverse correlation, while *VIM* was positively correlated with immune cell abundance. Stroma-adjusted epithelial genes also showed a more complex relationship to immune cell content: *EPCAM* was modestly positively correlated with immune cells, *ESRP1* was not significantly correlated, and both *ESRP2* and *CDH1* were moderately inversely correlated (Fig. 4A). We further profiled these associations by breaking the analysis up by PAM50 subtype. *COL1A1*, *FN1* and *CDH1* were all consistently negatively correlated with immune cell content, while the other markers did not exhibit consistent directional associations (Fig. 4B, Supplemental Table 5).

These results suggested that a simple relationship between EMT and immune cell content was unlikely. However, the specific association of stroma-corrected *COL1A1* expression with immune cell content was consistent with that observed for *ZEB1*. To further evaluate this correspondence, we calculated the partial correlation coefficients for *COL1A1* with xCELL estimates of cell types (Fig. 4C, Supplemental Table 6). While *COL1A1* was inversely associated with immune cell abundance, the cell types which showed the strongest associations were generally different from those associated with *ZEB1*. Both *ZEB1* and *COL1A1* exhibited a consistent inverse association with dendritic cell abundance (Fig. 4D, Supplemental Tables 4 and 6). However, *COL1A1* was strongly inversely correlated with CD4+ memory T-cells, a relationship *ZEB1* did not share. Similarly, *ZEB1* was significantly inversely correlated with Th1 cell and macrophage abundance, while *COL1A1* exhibited a much weaker association with both cell types (Fig. 4C,E). As a result, we concluded that the relationships of *ZEB1* and *COL1A1* expression to antitumor immunity are at least partially independent.

### Derivation of a high confidence stroma-corrected ZEB1 expression signature

**Figure 5:**
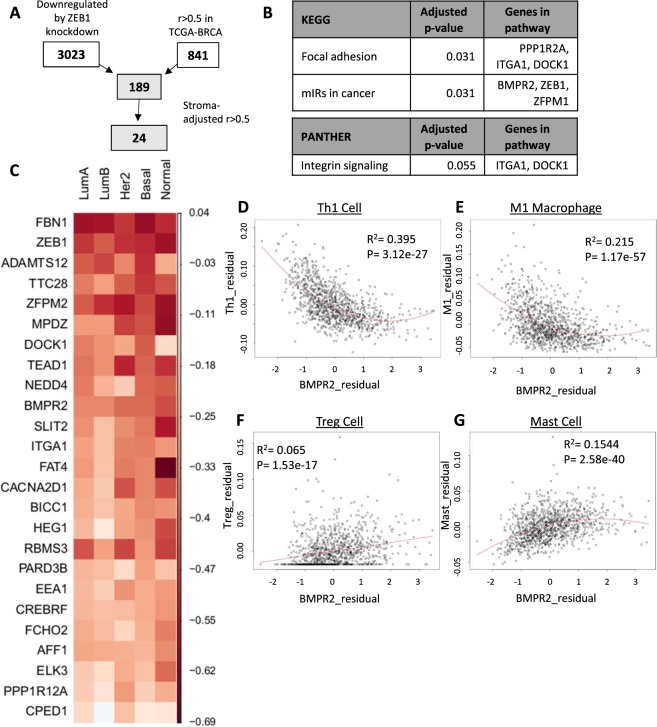
Stroma-adjusted ZEB1 transcriptional program is associated with decreased immune cell abundance in breast tumors. (**A**) Flowchart describing method for deriving ZEB1 transcriptional program in breast cancer. (**B**) Pathway analysis for 24-gene ZEB1 program. (**C**) Heatmap illustrating the partial correlation coefficients of ZEB1 program genes and immune content, adjusted for stromal content. (**D**–**G**) Residual plots for stroma-adjusted BMPR2 expression and Th1 (**D**), M1 macrophage (**E**), T-regulatory (**F**), and mast (**G**) cell content.

Since our results indicated that a general association between EMT-related gene expression and reduced immune cell activity was unlikely, we hypothesized that *ZEB1* may specifically regulate the immune response by activating the expression of immune-modulating genes. To identify a set of high-confidence *ZEB1*-regulated genes, an integrative transcriptomic pipeline was used, as illustrated in Fig. 5A: First, a previous study in which *ZEB1* was knocked down with shRNA in the MDA-MB-231 cell line and analyzed for differential gene expression was identified^35^. MDA-MB-231 is a mesenchymal breast cancer cell with high expression of *ZEB1*, providing an excellent model for studying *ZEB1* transcriptional activity in a breast cancer model^35^. This data was reanalyzed to identify a set of 3023 genes which were significantly (FDR < 0.05, log(fold change) <−2) downregulated by knockdown of *ZEB1*. Next, the set of genes which were highly correlated with *ZEB1* in the breast cancer TCGA dataset was curated with cBioPortal (Spearman r >0.5, 823 genes)^18,19^. A union of these two gene sets identified 186 genes which were both highly associated with *ZEB1* expression and differentially expressed upon *ZEB1* knockdown. This overlap was highly significant by the hypergeometric test, confirming that *ZEB1*-correlated genes are likely to be regulated by *ZEB1* transcriptional activity (1.5-fold enrichment, p = 4.20 × 10^−09^). The correlation between each gene and *ZEB1* was then adjusted by stromal score using partial correlation analysis. This approach identified 24 genes which remained significantly positively correlated with *ZEB1* (partial r > 0.5) (Supplemental Fig. 4). Pathway analysis of this gene set identified significant enrichment for focal adhesion and integrin signaling, as well as for genes targeted by cancer-associated microRNAs (Fig. 5B).

After deriving the *ZEB1* transcription program, we evaluated the association between the individual genes within the signature and stromal cell infiltration. As expected, all genes exhibited a significant positive association with stromal infiltration (Table 2). We then adjusted the expression of each gene by stromal score and examined the association between measures of immune activity. We found that all 24 genes were significantly inversely correlated with ESTIMATE immune score after adjustment for stromal score (average r = −0.21, range −0.09 to −0.55) (Table 2). As before, this association was then evaluated in the context of PAM50 subtypes. All stroma-adjusted genes except CPED1 showed a consistent inverse association with immune cell abundance, although not all the partial correlation coefficients reached significance (Fig. 5C).

**Table 2:** The ZEB1 transcriptional program is inversely associated with immune activity in breast tumors.

Gene	Stromal score correlation	p.Value	Adj. Correlation	p.value
HEG1	0.842	5.81e-296	−0.238	1.22E-15
RBMS3	0.744	1.99e-193	−0.381	4.40E-39
FBN1	0.843	3.83e-296	−0.584	4.10E-101
ZFPM2	0.803	3.83e-296	−0.462	5.42E-59
FAT4	0.735	2.07e-186	−0.318	3.35E-27
SLIT2	0.697	3.22e-160	−0.325	2.04E-28
ELK3	0.666	2.20e-141	−0.208	3.14E-12
BICC1	0.736	4.43e-187	−0.266	3.57E-19
CPED1	0.715	7.40e-172	−0.098	0.001126
ADAMTS12	0.624	4.08e-119	−0.439	9.89E-53
ITGA1	0.616	2.24e-115	−0.292	5.28E-23
CACNA2D1	0.522	1.90e-77	−0.286	4.38E-22
BMPR2	0.453	1.83e-56	−0.366	5.18E-36
TTC28	0.495	7.43e-69	−0.373	1.49E-37
NEDD4	0.415	6.21e-47	−0.338	9.59E-31
TEAD1	0.359	1.15e-34	−0.385	5.11E-40
EEA1	0.322	8.14e-28	−0.235	2.78E-15
CREBRF	0.319	2.48e-27	−0.241	5.42E-16
PARD3B	0.342	1.57e-31	−0.230	1.20E-14
FCHO2	0.325	1.87e-28	−0.246	1.36E-16
AFF1	0.269	1.13e-19	−0.265	4.45E-19
DOCK1	0.315	1.20e-26	−0.343	1.36E-31
PPP1R12A	0.269	1.37e-19	−0.182	1.37E-09
MPDZ	0.269	1.19e-19	−0.354	1.05E-33

As we hypothesized that genes within the *ZEB1* transcriptional signature might be responsible for the observed inverse relationship to immune activity, a literature review was performed to identify known modulators of the immune response within the gene set. Of the 24 genes, *bone morphogenic protein receptor 2* (*BMPR2*) was most consistently associated with immune activity in the literature. Inactivation or decreased expression of *BMPR2* has a well-characterized association with familial pulmonary arterial hypertension, an effect which may be due to the role of *BMPR2* in suppressing the response to TNF-mediated GM-CSF release^36,37^. *BMPR2* inactivation has also been shown to increase intratumoral inflammation in mouse models of breast cancer^38^.

Since *BMPR2* has a well-demonstrated association with reducing inflammation both physiologically and in models of breast cancer, we examined the specific associations between *BMPR2* expression and the xCELL-determined abundance of cell types. Like *ZEB1*, *BMPR2* was strongly negatively correlated with both Th1 cells and M1 macrophages, supporting an association with reduced pro-inflammatory cells within breast tumors (Fig. 5D,E, Supplemental Table 7). *BMPR2* exhibited a more robust positive association with T-regulatory cell abundance than *ZEB1* (Fig. 5F). Like *ZEB1*, *BMPR2* was also positively correlated with mast cell infiltration (Fig. 5G). These findings confirmed that the *ZEB1* transcriptional signature shares an overall inverse association with antitumor immunity and suggests that genes contained within this signature may be functionally responsible for this association.

### ZEB1 expression and ZEB1 transcription activity is associated with worse overall survival in breast cancer

**Figure 6:**
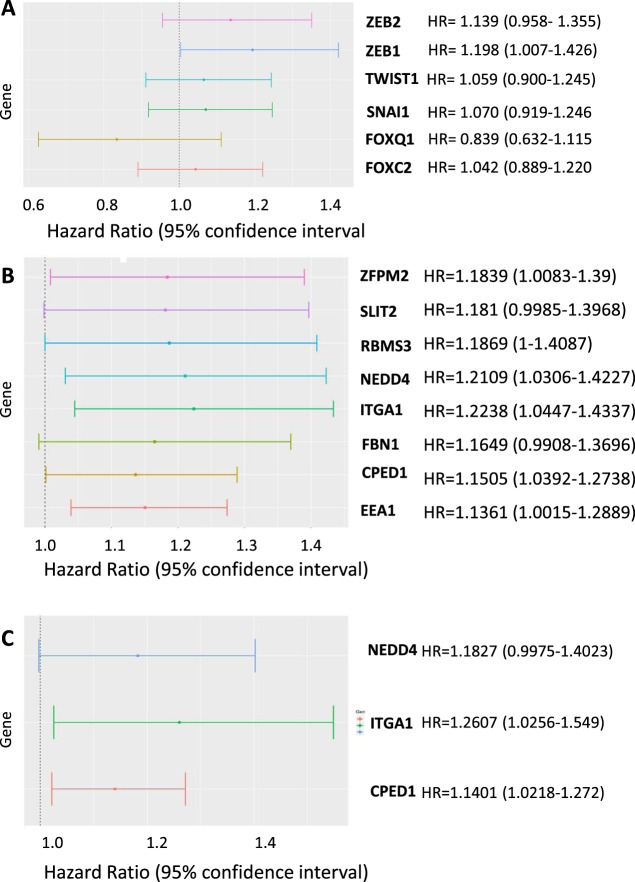
ZEB1 and the ZEB1 transcriptional program are associated with worse overall survival in breast cancer. (**A**) Forest plot of hazard ratios for multiple EMT-TFs in breast cancer, individually included in a multivariate Cox proportional hazards model. (**B**) Forest plot of 8 significantly prognostic genes within the ZEB1 transcriptional signature individually included in the multivariate Cox model. (**C**) Three genes within signature which remain significantly prognostic after adjusting for stromal score.

Finally, we evaluated the associations of *ZEB1*, other EMT-TFs and the *ZEB1* program genes with overall survival in breast cancer patients. We observed that no EMT-TF was significantly associated with survival when included in a univariate Cox regression model. However, when the known prognostic factors of pathologic stage, the age of the patient at diagnosis and estrogen receptor status were included in the model, only *ZEB1* was a significant independent predictor of overall survival (HR 1.21, 95% CI 1.00–1.42, p = 0.045) (Fig. 6A). Next, we included each *ZEB1* program gene independently in the same multivariate model. We found that increased expression of 8 of 24 genes were significantly associated with decreased survival time (Fig. 6B).

Finally, we examined how adjusting gene expression by stromal score affected the association with overall survival. Stromal score itself was not a significant independent prognostic factor (data not shown). When adjusted for stromal score, three genes (*ITGA1*, *NEDD4*, *CPED1*) remained significant independent prognostic factors, although all eight genes still exhibited a general trend towards an association with worse overall survival (Fig. 6C). This decrease in the number of genes reaching statistical significance was due to widening of the confidence interval for the residuals, rather than a directional effect. Together, these data indicate that expression of the *ZEB1* transcriptional program is associated with reduced overall survival in breast cancer.

## Discussion

This study demonstrates that stromal cells within tumors can significantly affect the statistical evaluation of associations between gene expression levels, tumor immune activity, and patient outcomes. By correcting for stromal cell abundance, our analyses demonstrated that previous attempts to study EMT using bulk tumor sequencing data were likely confounded. Our results also suggest that EMT-associated transcription factors and marker genes cannot be treated as interchangeable representatives of a common program. In fact, these results show a diverse range of associations between EMT-associated genes and both tumor stromal and immune cell content, indicated that each gene may have a unique functional association with the tumor microenvironment.

Previous studies have reported a significant association between EMT and immune activity in several cancers without accounting for the gene expression contribution of the stromal compartment^11,13^. We observed that the association between several EMT markers and immune cell infiltration were significantly influenced by stromal content. This suggests that previous findings linking EMT to altered immune activity may have been confounded by stroma, a possibility that has been suggested by other groups^39^. It was also shown that only some EMT markers have strong inverse associations with immune cell abundance, indicating that prior attempts to generalize an association between EMT-affiliated gene expression with the tumor microenvironment were misguided.

When adjusted for stromal cell abundance, *ZEB1* gene expression is significantly inversely correlated with multiple measures of the immune response. This effect was strongest with *ZEB1* among the several EMT-TFs examined. These results suggest that *ZEB1* transcriptional activity in tumor cells may modulate the antitumor immune response. However, as this study relied on retrospective and correlative data, it is not yet possible to determine the causal relationship implied by this association.

Importantly, this study identified a novel inverse association between the transcription factor *ZEB1* and immune activity in multiple cancer types. By deriving a set of *ZEB1* activated and correlated genes, we demonstrated that *ZEB1* transcriptional activity is also correlated with decreased tumor immune activity and provided a set of potential functional regulators of the tumor immune response for further investigation.

In addition to *BMPR2*, several genes within the stroma-adjusted *ZEB1* transcriptional program have been characterized as modulators of immune activity. Fibrillins, including the *ZEB1* program gene fibrillin 1 (*FBN1*), are critical regulators of immune activity^40^. Inactivating mutations in the cell-matrix interaction domain of *FBN1* have been demonstrated to lead to significantly increased pro-inflammatory cell infiltration and fibrosis in the skin^41^. Another member of the *ZEB1* transcriptional program, the *ROBO* receptor ligand *SLIT2*, has been demonstrated to inhibit the response of antigen-presenting cells to allergic skin reactions^42^. SLIT-ROBO signaling has also been implicated in the inhibition of leukocyte chemotaxis^43^. Beyond the set of genes identified by this study, *ZEB1* has also been shown to directly regulate the expression of IL-6 and other cytokines by breast cancer cells to promote accumulation of myeloid-derived suppressor cells in a mouse model of breast cancer^44^. As IL-6 has also been shown to recruit mast cells to the tumor microenvironment, *ZEB1*-mediated IL-6 expression may explain the significant positive association we observed between *ZEB1* expression and the abundance of intratumoral mast cells^29^. *ZEB1* has also been shown experimentally to regulate *PD-L1* expression in breast tumors, although our analysis did not identify a significant correlation between *ZEB1* and *PD-L1* expression^45^. Inhibiting *ZEB1*-mediated immune suppression, either directly or by inhibiting ZEB1-regulated immune modulators like *BMPR2*, may be a therapeutic approach to promote antitumor immune activity in breast cancer.

## Methods

### Expression and clinical data

Primary tumor RNAseqV2 expression and clinicopathologic data for the TCGA breast, lung, prostate, pancreatic and colon adenocarcinoma cohorts was downloaded from the cBioPortal in the form of z-scores^19^. Data was combined by comparison of sample ID and filtered to include primary tumor samples only. For the METABRIC cohort, microarray expression data in the form of normalized log intensity level was downloaded from the cBioPortal^33^. Microarray data from Lehmann *et al*. (E-MTAB-3482) for MDA-MB-231 with knockdown of either GFP (control) or *ZEB1* were downloaded from https://www.ebi.ac.uk/arrayexpress ^35^.

### Measures of tumor purity and immune cell content

ESTIMATE (Estimation of STromal and Immune cells in MAlignant Tumor tissues using Expression data), which estimates stromal and immune cell content in tumors by using ssGSEA to rank samples on the expression of two 141-gene sets, was used to determine tumor purity. ESTIMATE stromal and immune scores for all TCGA datasets were obtained from the *ESTIMATE* online platform and matched to gene expression and clinical data from cBioPortal using sample ID codes^46^. For METABRIC data, ESTIMATE scores were generated using the *estimate* R package^20^. Immune activity scores were obtained from the TIP server^30^. Stromal and immune cell IHC percent estimates were obtained for the TCGA breast cancer dataset using Firebrowse and formatted with the *psichomics* R package^47^. Patients were split into two groups by median *ZEB1* expression and evaluated by Wilcoxon rank-sum test to determine if the two groups exhibited significant differences in IHC-measured purity.

For cell-type specific analyses, the xCELL algorithm was used to generate estimates for the relative proportions of 64 cell types^25^. xCELL uses a deconvolution-based GSEA approach to determine the proportion of each cell type. xCELL scores for the TCGA dataset were downloaded from the xCELL server (http://xcell.ucsf.edu/). For immune activity profiling, the Tumor ImmunoPhenotype (TIP) pipeline was used^30^. This approach uses a similar, GSEA-based approach to evaluate the relative activity of the seven “steps” of the immune cycle within bulk tumor samples. TIP scores for the BRCA TCGA subset were downloaded from the online TIP server (http://biocc.hrbmu.edu.cn/TIP/).

### Regression analyses

The association between gene expression, measures of tumor purity and clinical characteristics was performed using both linear models and partial correlations. Univariable analysis of variance (ANOVA) was used to test the significance each predictor variable. Multivariable ANOVA was used to identify a parsimonious set of predictor variables The *ppcor* R package was used to generate Spearman’s partial correlation coefficients^22^. The partial correlation coefficients were illustrated by plotting the residuals of the final parsimonious model excluding the variable of interest versus the residuals of a model with the variable of interest as the response versus the same parsimonious set of predictors (excluding the variable of interest). Linear models were used to generate the coefficient of determination for each association. As many of the relationships are quadratic in nature, the r^2^ value on plots may not precisely correspond to r values determined by partial correlation analysis.

### Derivation of a *ZEB1* transcriptional signature

First, microarray data from Lehmann *et al*. were processed and analyzed for differential expression using the Transcriptome Analysis Console (TAC) from ThermoFisher. The set of significantly downregulated genes (FDR < 0.05, log(Fold change) <−2) was obtained. Next, genes which were significantly (Spearman r >0.5) correlated with *ZEB1* in the breast cancer TCGA dataset were identified and downloaded. The two gene sets were compared for intersection, with 186 genes contained in both sets. Finally, the BRCA-TCGA expression data for these genes in the breast cancer dataset was downloaded. The partial correlation for each gene with *ZEB1* was computed, adjusting for the stromal cell content using the *ppcor* package. The overlap between *ZEB1*-regulated and -correlated genes was tested for significance using the hypergeometric test.

### Survival analyses

Survival analysis was conducted with the *survival* package in R^48^. Univariate and multivariate Cox proportional hazards models were generated. The multivariate model included patient stage, age at diagnosis and estrogen receptor status, as these were all significant independent clinical predictors of overall survival. Plots representing the hazard ratio and 95% confidence interval were generated with *ggplot2* in R^49^.

## Supplementary information

Supplementary Information

Supplementary Dataset 1

## Data Availability

All data from the TCGA and METABRIC was downloaded by either cBioPortal or Firehose. Microarray data from Lehmann *et al*. was obtained from GEO. All final outputs from our analyses are available as csv files upon request.
