# Supplementary material for: A stroma-corrected ZEB1 transcriptional signature is inversely associated with antitumor immune activity in breast cancer
Source: Sci Rep. 2019 Nov 28;9:17807. doi: 10.1038/s41598-019-54282-z (PMC6882801; doi:10.1038/s41598-019-54282-z)
Supplement: Supplementary file 1 — Supplementary Information [file 41598_2019_54282_MOESM1_ESM.pdf]

Supplementary Information

A stroma-corrected ZEB1 transcriptional signature is inversely associated with antitumor immune activity in breast cancer

C James Block[1,2], Gregory Dyson[1], Ion John Campeanu[1], Donovan Watza[1,2], Manohar Ratnam[1] and Guojun Wu[1;] *

1. Barbara Ann Karmanos Cancer Institute, Department of Oncology,, Wayne State University School of Medicine, 4100 John R, Detroit MI

2. MD/PhD Program, Wayne State University School of Medicine, 320 E Canfield St., Detroit MI

*Corresponding author:

Guojun Wu, Ph.D.

Associate Professor

Molecular Therapeutics Program

Karmanos Cancer Institute

Department of Oncology

Wayne State University School of Medicine

4100 John R

HWCRC, Room 824

Detroit, MI 48201

(313) 576-8349 (O)

Email: wugu@karmanos.org

Supplemental Figures

- Supplemental figure 1: Example of confounding by stromal cell abundance
- Supplemental figure 2: Correlation heatmap for 64 xCELL cell subtypes in the TCGA breast cancer dataset
- Supplemental figure 3: Partial correlation coefficients between ZEB1 and cytokines and correlation results for ZEB1 and stromal and immune ESTIMATE scores in METABRIC dataset
- Supplemental figure 4: List of 24 ZEB1 genes and their partial correlation with ZEB1 expression
- Supplemental table 1: Correlation coefficients for EMT-TFs with ESTIMATE scores in prostate, lung, colorectal, pancreatic ductal and breast adenocarcinomas
- Supplemental table 2: Partial correlation coefficients for EMT-TFs with ESTIMATE immune score, adjusted for stromal score, across the 5 cancer types
- Supplemental table 3: Partial correlation coefficients for EMT-TFs with ESTIMATE immune score, adjusted for stromal score, across PAM50 breast cancer subtypes
- Supplemental table 4: Partial correlation coefficients for ZEB1 with xCELL-estimated cell types, adjusted for stromal score, in all breast cancer samples
- Supplemental table 5: Partial correlation between EMT markers and ESTIMATE immune score, adjusted for stromal score, in PAM50 breast cancer subtypes
- Supplemental table 6: Partial correlation coefficients for COL1A1 and xCELL-estimated cell types, adjusted for stromal score, in breast cancer dataset
- Supplemental table 7: Partial correlation coefficients for BMPR2 and xCELL-estimated cell types, adjusted for stromal score, in breast cancer dataset
- Supplemental data 1: ESTIMATE scores and ZEB1 expression from METABRIC dataset

Supplemental figure 1: Bulk tumor sequencing results can be affected by abundance of non-cancer cells present in sample

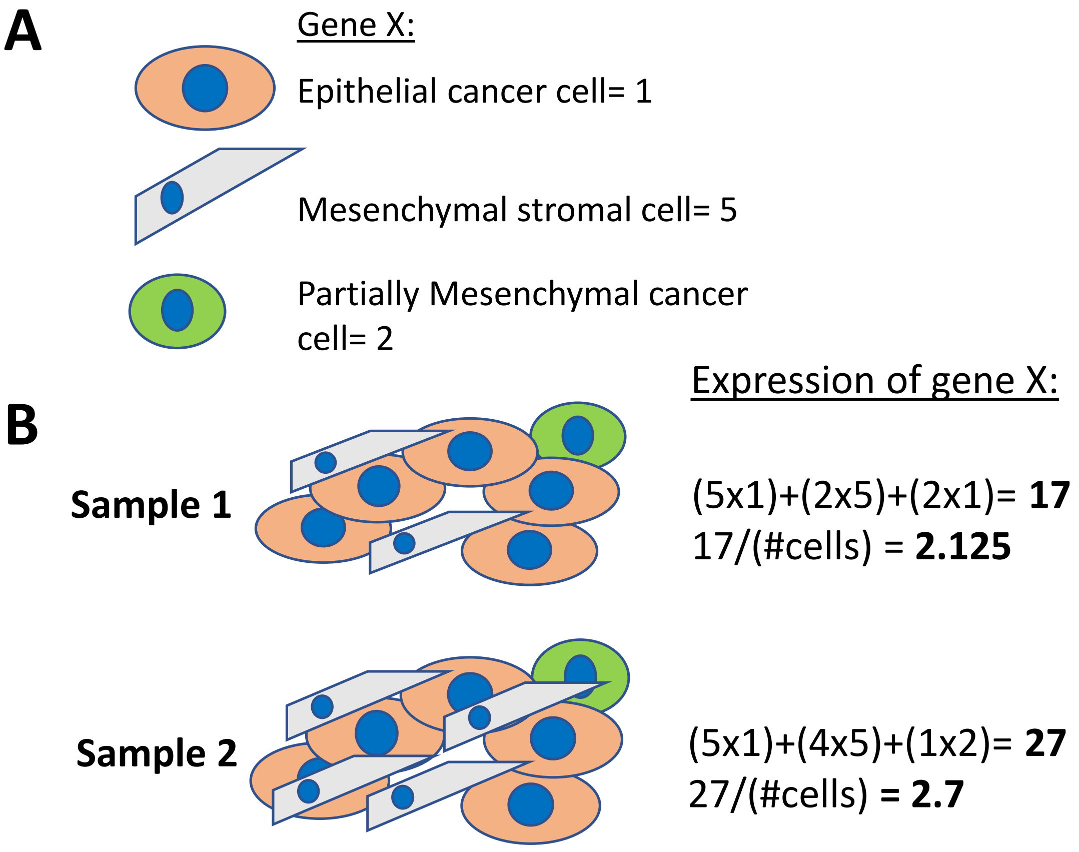

Supplemental figure 2: Correlation heatmap for 64 xCELL cell subtypes in the TCGA breast cancer dataset

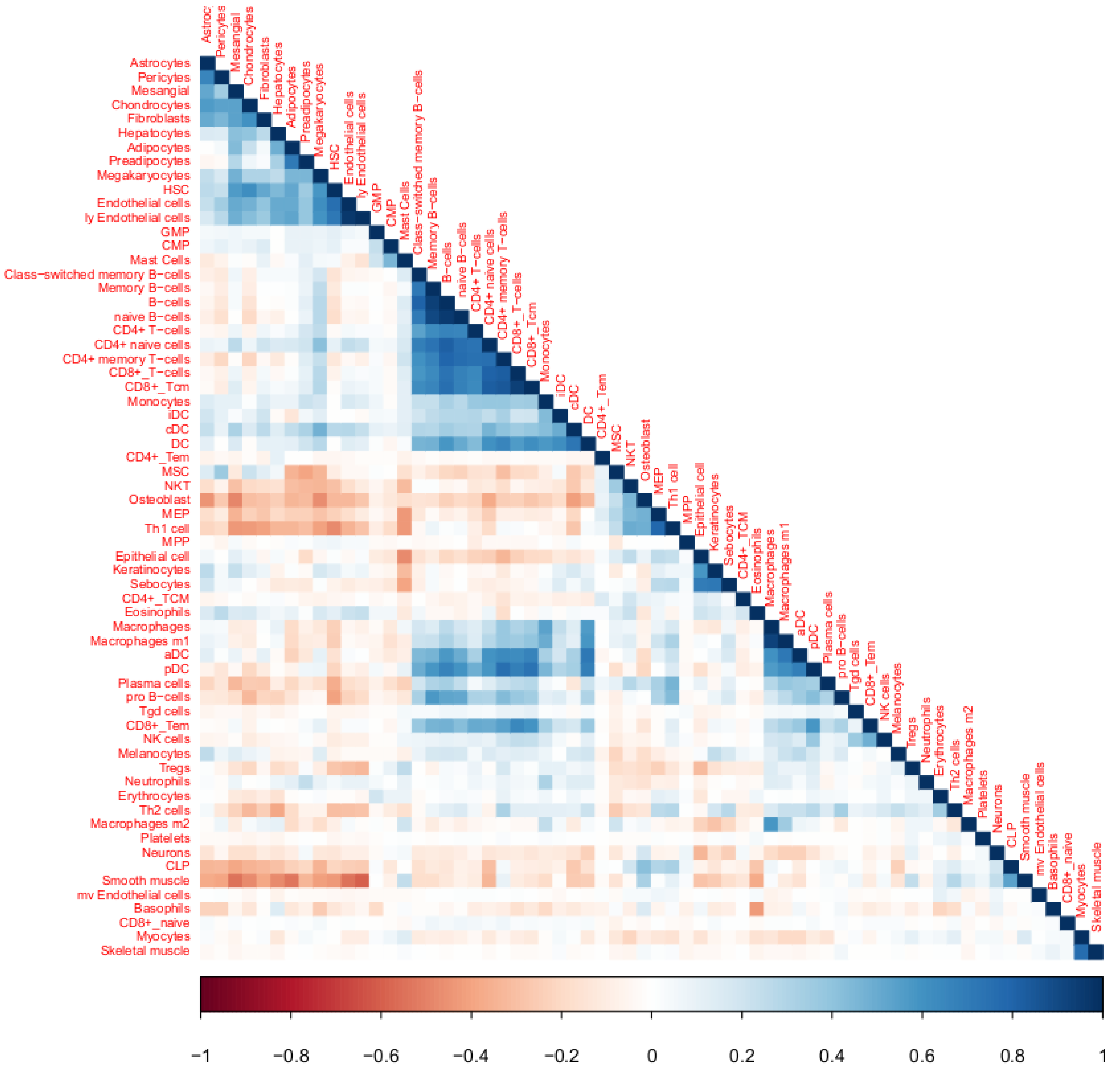

Supplemental figure 3: Partial correlation coefficients between ZEB1 and cytokines and correlation results for ZEB1 and stromal and immune ESTIMATE scores in METABRIC dataset

A

Gene	Partial r	P-value
**CCL2**	-0.367	1.03e-35
**CCL4**	-0.396	1.02e-41
**IFNG**	-0.305	1.17e-24
**IL6**	-0.173	9.79e-09
**IL18**	-0.403	2.00e-43
**IL1A**	-0.200	3.45e-11
**IL1B**	-0.236	4.67e-15
**MIF**	-0.394	3.16e-41
**TNF-α**	-0.306	9.21e-25

B

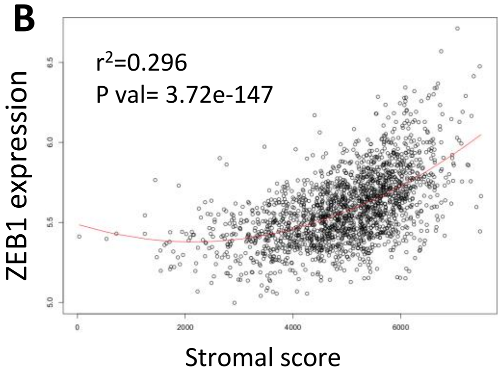

C

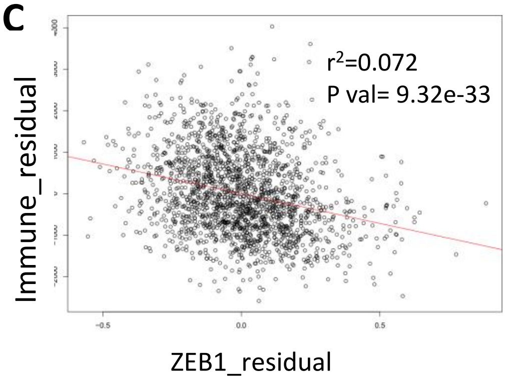

Supplemental figure 4: List of 24 ZEB1 genes and their partial correlation with ZEB1 expression

Gene	Adj. correlation	p.Value
RBMS3	0.6436	9.21e-129
SLIT2	0.6327	3.54e-123
ELK3	0.6144	2.43e-114
BMPR2	0.6138	4.77e-114
FAT4	0.6033	3.06e-109
AFF1	0.5743	7.78E-97
HEG1	0.5684	1.81E-94
CREBRF	0.5665	1.03E-93
EEA1	0.5577	2.87E-90
CACNA2D1	0.5567	6.65E-90
NEDD4	0.5326	4.94E-81
ZFPM2	0.5298	4.74E-80
PPP1R12A	0.5288	1.05E-79
ITGA1	0.5223	1.80E-77
FCHO2	0.518	5.25E-76
TEAD1	0.517	1.10E-75
MPDZ	0.5155	3.74E-75
FBN1	0.5111	1.00E-73
CPED1	0.511	1.07E-73
BICC1	0.5102	2.05E-73
TTC28	0.5088	5.88E-73
ADAMTS12	0.5087	6.49E-73
PARD3B	0.4994	6.02E-70
DOCK1	0.4959	7.63E-69

Supplemental table 1: Correlation coefficients for EMT-TFs with ESTIMATE scores in prostate, lung, colorectal, pancreatic ductal and breast adenocarcinomas

Gene	PST_Stromal_score	PST_Immune_score	PDAC_Stromal_score	PDAC_Immune_score	LUAD_Stromal_score	LUAD_Immune_score	BRCA Stroma	BRCA_Immune_Score
ZEB1	0.832879	0.429296	0.833535	0.568321	0.776091	0.445022	0.826799	0.2419
ZEB2	0.820438	0.530047	0.888006	0.69209	0.856755	0.724489	0.850903	0.59872
TWIST1	-0.03577	-0.1288	0.476946	0.28751	0.421889	0.19746	0.553712	0.30574
SNAI1	0.560614	0.480513	0.658894	0.576692	0.41372	0.247347	0.288102	0.33268
SNAI2	0.394624	0.280261	0.580559	0.390483	0.678873	0.297131	0.684729	0.2403
FOXQ1	0.160881	0.255403	-0.37979	-0.27641	0.080821	0.122909	0.276148	0.16851
FOXC2	0.5532	0.355399	0.227877	0.193776	0.418709	0.090038	0.277095	0.08248

Supplemental table 2: Partial correlation coefficients for EMT-TFs with ESTIMATE immune score, adjusted for stromal score, across the 5 cancer types

Cancer	ZEB1	ZEB2	TWIST1	SNAI1	SNAI2	FOXQ1	FOXC2
BRCA	-0.50358	0.253774	-0.014	0.215117	-0.2526	0.013444	-0.0947
COAD	-0.34128	0.128321	-0.26541	-0.17419	-0.14963	-0.29026	-0.27201
LUAD	-0.19114	0.363776	-0.1397	-0.05531	-0.31616	0.093031	-0.29882
PDAC	-0.34529	-0.11717	-0.1981	0.091615	-0.17452	0.061408	0.014436
PST	-0.35383	-0.07966	-0.14345	0.158915	0.014043	0.201985	-0.04016

Supplemental table 3: Partial correlation coefficients for EMT-TFs with ESTIMATE immune score, adjusted for stromal score, across PAM50 breast cancer subtypes

Gene	LumB	LumA	Basal	Her2	Normal
ZEB1	-0.3539	-0.41772	-0.49194	-0.44179	-0.50409
SNAI2	-0.1138	-0.02945	-0.17519	-0.47511	-0.25015
SNAI1	0.108269	0.220112	0.232696	0.184907	-0.1139
FOXC2	0.042907	0.106844	0.06055	-0.15521	0.096256
FOXQ1	0.075795	0.183034	0.105406	-0.00317	0.126775
ZEB2	0.377515	0.322586	0.195036	0.332377	0.211155
TWIST1	0.039174	0.018094	0.151741	-0.20625	0.247511

Supplemental table 4: Partial correlation coefficients for ZEB1 with xCELL-estimated cell types, adjusted for stromal score, in all breast cancer samples

ZEB1	Partial Corr	pval
Macrophages m1	-0.5933	2.05E-103
Th1 cell	-0.5489	9.08E-86
Macrophages	-0.4968	3.31E-68
aDC	-0.4563	1.74E-56
MEP	-0.4393	4.89E-52
Plasma cells	-0.4379	1.13E-51
DC	-0.4226	6.87E-48
Sebocytes	-0.3351	1.14E-29
Epithelial cell	-0.2883	4.68E-22
NKT	-0.2720	1.02E-19
Monocytes	-0.2676	4.09E-19
Memory B-cells	-0.2607	3.46E-18
pDC	-0.2497	9.07E-17
naive B-cells	-0.2357	4.68E-15
Osteoblast	-0.2212	2.12E-13
pro B-cells	-0.2130	1.61E-12
B-cells	-0.2094	3.88E-12
Class-switched memory B-cells	-0.1511	6.34E-07
Keratinocytes	-0.1401	3.93E-06
Melanocytes	-0.10954	0.000316555
MSC	-0.10765	0.000401542
CD4+_Tem	-0.10669	0.000452855
CD4+ memory T-cells	-0.10507	0.000553066
CD8+_Tcm	-0.09441	0.001923474
CD8+_T-cells	-0.08367	0.006006034
Astrocytes	-0.08153	0.00742877
Th2 cells	-0.08108	0.007761068
Macrophages m2	-0.07804	0.010409092
CD8+_Tem	-0.07444	0.014539708
Basophils	-0.06934	0.022870252
Hepatocytes	-0.06929	0.022961579
CD4+ T-cells	-0.06695	0.028011826
NK cells	-0.06443	0.034505329
GMP	-0.04204	0.168028482
CD4+ naive cells	-0.03898	0.201140865
Neutrophils	-0.03806	0.212015455
CD4+_TCM	-0.03424	0.261607575
Tgd cells	-0.0307	0.314144307
iDC	-0.02622	0.389997594
mv Endothelial cells	-0.02552	0.402814657
Eosinophils	-0.01204	0.693081069
Platelets	-0.00838	0.783464315
Pericytes	0.000339	0.991136737
Erythrocytes	0.014584	0.632586488
MPP	0.033338	0.274349631
CD8+_naive	0.03342	0.273165567
Fibroblasts	0.034014	0.264732489
CLP	0.042002	0.168382579
cDC	0.047795	0.116977656
ly Endothelial cells	0.053075	0.081685856
Chondrocytes	0.058788	0.053765093
Skeletal muscle	0.059059	0.052668747
Mesangial	0.09269	0.002327649
Endothelial cells	0.113868	0.000180591
Adipocytes	0.1214	6.52E-05
CMP	0.1251	3.82E-05
Preadipocytes	0.1296	1.98E-05
Megakaryocytes	0.1597	1.37E-07
Tregs	0.1771	4.91E-09
HSC	0.2525	3.98E-17
Smooth muscle	0.2961	3.09E-23
Myocytes	0.3536	4.50E-33
Neurons	0.3915	8.82E-41
Mast Cells	0.4001	1.13E-42

Supplemental table 5: Partial correlation between EMT markers and ESTIMATE immune score, adjusted for stromal score, in PAM50 breast cancer subtypes

Gene	LumB	LumA	Basal	Her2	Normal
CDH1	-0.1688	-0.19364	-0.11427	-0.1348	-0.17913
CDH2	-0.08832	-0.06009	-0.06878	0.072316	0.090324
COL1A1	-0.4326	-0.32244	-0.25639	-0.22687	-0.20695
EPCAM	0.114139	0.05518	0.012627	-0.06438	-0.08573
ESRP1	0.011803	-0.0013	-0.05972	0.230505	0.072966
ESRP2	-0.04816	-0.07487	0.010313	-0.02057	-0.11026
FN1	-0.34435	-0.33449	-0.22162	-0.0794	-0.3145
VIM	0.182202	0.097955	0.132772	-0.13085	0.065572

Supplemental table 6: Partial correlation coefficients for COL1A1 and xCELL-estimated cell types, adjusted for stromal score, in breast cancer dataset

Cell Type	Adj. Correlation	P.value
CD4+ memory T-cells	-0.51495	6.53E-74
DC	-0.43102	6.58E-50
B-cells	-0.41362	1.02E-45
pDC	-0.37938	3.65E-38
naive B-cells	-0.36692	1.25E-35
CD8+_Tcm	-0.34506	1.91E-31
CD8+_T-cells	-0.34213	6.55E-31
CD4+ naive cells	-0.33979	1.74E-30
cDC	-0.32168	2.52E-27
aDC	-0.31979	5.23E-27
Megakaryocytes	-0.28804	5.29E-22
Preadipocytes	-0.23434	6.88E-15
Macrophages	-0.23257	1.11E-14
Monocytes	-0.21542	9.19E-13
Melanocytes	-0.20931	4.07E-12
Macrophages m1	-0.20603	8.87E-12
Memory B-cells	-0.19836	5.22E-11
Skeletal muscle	-0.18844	4.67E-10
Mast Cells	-0.18467	1.04E-09
Endothelial cells	-0.1551	3.17E-07
Th2 cells	-0.15083	6.68E-07
CD4+ T-cells	-0.1503	7.32E-07
Class-switched memory B-cells	-0.14559	1.62E-06
Plasma cells	-0.14019	3.91E-06
Neutrophils	-0.12754	2.72E-05
Tregs	-0.11281	0.000208966
MPP	-0.11162	0.000243835
ly Endothelial cells	-0.1067	0.000454786
Smooth muscle	-0.09469	0.001873471
CD8+_naive	-0.07913	0.009416021
CD8+_Tem	-0.06885	0.023924152
pro B-cells	-0.06306	0.038619759
CLP	-0.05343	0.079808664
NK cells	-0.04499	0.140249406
Basophils	-0.03656	0.230845683
Erythrocytes	-0.0333	0.2751507
Myocytes	-0.02294	0.452299856
Adipocytes	-0.02237	0.46359069
Keratinocytes	-0.02	0.512205307
Neurons	-0.01825	0.549801489
HSC	-0.01589	0.602507669
iDC	-0.01124	0.712648194
Tgd cells	0.001461	0.96181339
Macrophages m2	0.006312	0.836161582
Platelets	0.010438	0.732353027
mv Endothelial cells	0.02639	0.38714516
Mesangial	0.03219	0.291445799
CD4+_Tem	0.044451	0.145082687
Hepatocytes	0.048213	0.113975125
CMP	0.068451	0.024743037
GMP	0.078557	0.009941752
Sebocytes	0.138139	5.42E-06
CD4+_TCM	0.142262	2.80E-06
Fibroblasts	0.14961	8.24E-07
Th1 cell	0.154152	3.75E-07
MEP	0.161929	9.25E-08
Eosinophils	0.163423	7.02E-08
Osteoblast	0.213036	1.65E-12
Epithelial cell	0.246363	2.43E-16
NKT	0.267268	4.68E-19
Chondrocytes	0.28403	2.05E-21
Astrocytes	0.321778	2.42E-27
Pericytes	0.441872	1.20E-52
MSC	0.590298	5.06E-102

Supplemental table 7: Partial correlation coefficients for BMPR2 and xCELL-estimated cell types, adjusted for stromal score, in breast cancer dataset

Cell type	pcor	pval
Th1 cell	-0.61295	4.18E-112
MEP	-0.51414	1.03E-73
Macrophages m1	-0.43199	3.42E-50
Plasma cells	-0.39947	1.59E-42
NKT	-0.386	1.36E-39
aDC	-0.33871	2.55E-30
Macrophages	-0.33515	1.11E-29
MSC	-0.29629	2.90E-23
Osteoblast	-0.29302	9.11E-23
DC	-0.2495	9.55E-17
Epithelial cell	-0.24545	3.06E-16
Memory B-cells	-0.2321	1.22E-14
Sebocytes	-0.18736	5.77E-10
pro B-cells	-0.17097	1.64E-08
Hepatocytes	-0.16602	4.25E-08
Class-switched memory B-cells	-0.16438	5.79E-08
Monocytes	-0.13513	8.58E-06
Basophils	-0.10796	0.000386229
pDC	-0.101	0.000902948
CD4+_Tem	-0.09483	0.001835586
naive B-cells	-0.09297	0.00225616
B-cells	-0.08526	0.005113967
CD4+ T-cells	-0.07952	0.009035841
ly Endothelial cells	-0.07849	0.009974558
Macrophages m2	-0.07553	0.013158285
Eosinophils	-0.06476	0.033571677
CLP	-0.06203	0.041821215
CD4+_TCM	-0.05203	0.087855163
CD8+_Tem	-0.05057	0.097203976
mv Endothelial cells	-0.04612	0.130420294
Pericytes	-0.04211	0.167287754
Keratinocytes	-0.04057	0.183346337
CD8+_T-cells	-0.03211	0.292397778
CD8+_Tcm	-0.02932	0.336381704
Fibroblasts	-0.02188	0.473123304
Endothelial cells	-0.01978	0.516697723
GMP	-0.01884	0.536840189
CD4+ naive cells	-0.00275	0.928241141
Platelets	0.000279	0.992699684
Astrocytes	0.016407	0.590681617
NK cells	0.018267	0.549286511
Chondrocytes	0.019105	0.531111341
Tgd cells	0.020913	0.492965703
Melanocytes	0.023316	0.444637328
CD8+_naive	0.024142	0.428672804
Adipocytes	0.025038	0.411731745
CD4+ memory T-cells	0.025219	0.408349469
Neutrophils	0.028893	0.343492237
MPP	0.035929	0.238747009
Skeletal muscle	0.038113	0.211373742
iDC	0.054166	0.075591982
Erythrocytes	0.059578	0.050621771
Mesangial	0.067277	0.027257607
Th2 cells	0.07868	0.009792028
Preadipocytes	0.082838	0.006527011
CMP	0.100733	0.000931597
HSC	0.133195	1.16E-05
cDC	0.219496	3.24E-13
Megakaryocytes	0.235734	4.58E-15
Neurons	0.325954	4.46E-28
Tregs	0.329247	1.20E-28
Smooth muscle	0.359237	3.75E-34
Mast Cells	0.40088	7.68E-43
Myocytes	0.505718	5.37E-71
